# Microenvironment of Tumor-Draining Lymph Nodes: Opportunities for Liposome-Based Targeted Therapy

**DOI:** 10.3390/ijms151120209

**Published:** 2014-11-05

**Authors:** Siddarth Chandrasekaran, Michael R. King

**Affiliations:** Department of Biomedical Engineering, Cornell University, Ithaca, NY 14853, USA; E-Mail: sc2389@cornell.edu

**Keywords:** tumor microenvironment, liposomes, nanomedicine, metastasis, tumor-draining lymph nodes

## Abstract

The World Health Organization (WHO) recently reported that the total number of global cancer cases in 2013 reached 14 million, a 10% rise since 2008, while the total number of cancer deaths reached 8.2 million, a 5.2% increase since 2008. Metastasis is the major cause of death from cancer, accounting for 90% of all cancer related deaths. Tumor-draining lymph nodes (TDLN), the sentinel nodes, are the first organs of metastasis in several types of cancers. The extent of metastasis in the TDLN is often used in disease staging and prognosis evaluation in cancer patients. Here, we describe the microenvironment of the TDLN and review the recent literature on liposome-based therapies directed to immune cells within the TDLN with the intent to target cancer cells.

## 1. Introduction

Tumor-draining lymph nodes (TDLN) are the first organs of metastases in malignant melanomas [1], most carcinomas [2] and some sarcomas [3]. The presence of cancer cells in lymph nodes has been used as an important prognosticator in cancer patients [4,5,6,7,8]. Lymph nodes (LN) are lymphoid organs composed of different types of immune cells strategically positioned throughout the body and they play an important role in the immune response. Despite the presence of cells that can induce an anti-tumor immune response, the TDLN often act as a mediator of cancer cells leading to distant organ metastases. T-cells, B-cells, natural killer (NK) cells and antigen presenting cells (APC) are the majority of the immune cells present in the LN. Dendritic cells (DC) and macrophages comprise the major types of APC present in the LN [9]. Lymphatic vessels channel the fluid derived from the interstitial spaces of most tissues (lymph) containing important biochemical information (such as proteins and macromolecules) regarding the local immune signature. LN are structures at which lymphatic vessels converge, giving an opportunity for lymph to interact with the resident immune cells to mediate an immune response. The immune system is composed of two separate yet interactive compartments: the innate and the adaptive immune systems. The innate immune system is the first line of defense that protects the body from pathogens that may cross physical barriers such as the skin and the mucosal layer [10]. It is composed of macrophages, eosinophils, basophils, neutrophils and granulocytes that can phagocytose pathogens based on their ability to recognize specific patterns on the pathogen surface [11]. The cells of the innate immune system can also secrete chemokines that can attract other immune cells such as natural killer (NK) cells that produce enzymes to kill pathogens. T- and B-cells mediate the adaptive immune response. They differ from cells of the innate immune response by their specificity and long-term memory capable of providing protection against a second encounter with the same pathogen [12]. The adaptive immune response can either be cellular (mediated by T-cells) or humoral (mediated by B-cells). To understand the homing of immune cells to LN and cellular traffic in LN, see Ref. [13].

**Figure 1:**
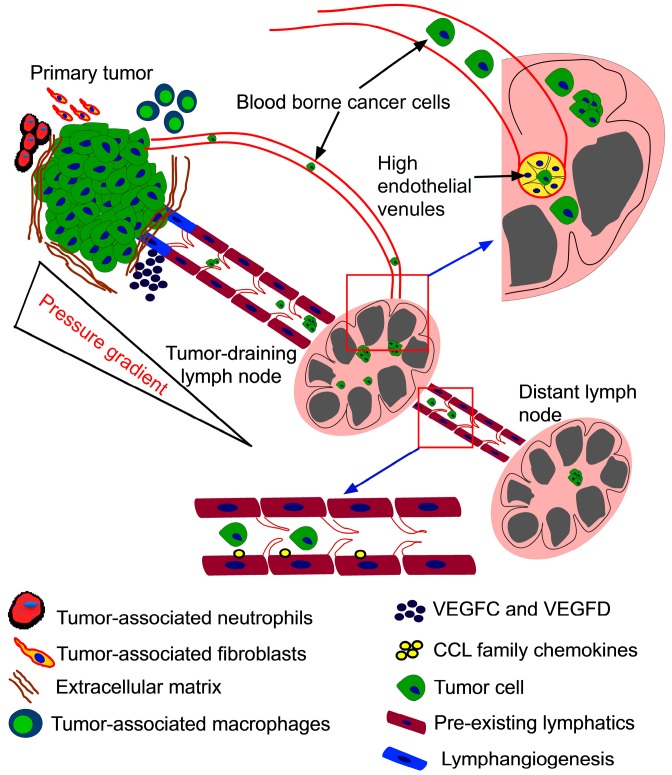
How do cancer cells reach the tumor-draining lymph nodes (TDLN)? The primary tumor microenvironment is regulated by the interactions between tumor cells, extracellular matrix (ECM), tumor-associated neutrophils and fibroblasts. These interactions induce the secretion of vascular endothelial growth factors C and D (VEGFC and VEGFD) that mediate the formation of new lymphatic capillaries around the primary tumor. Cells from the primary tumor travel through the lymphatics either as single cells or as clusters in response to the pressure gradient. Chemokine attractions facilitated by chemokine ligands (CCL) family chemokines secreted by endothelial cells and chemokine receptors (CCR) family receptors expressed on tumor cells can also facilitate the translocation of tumor cells through the lymphatic capillaries. The translocated cells get lodged in the TDLN to form micrometastases. Alternatively, cells from the primary tumor can enter the peripheral circulation and reach the TDLN through the high endothelial venules in the TDLN.

Most human cancers metastasize through the lymphatic system. It has been estimated that 80% of melanomas and carcinomas metastasize through the lymphatic system and 20% through blood vasculature and direct seeding [14]. The percentage of individuals with metastases in their LN at the time of diagnosis is significantly higher than metastases in other organs. For instance, 29%–37% of individuals with breast, colorectal and lung cancer are diagnosed with metastases in their LN [15]. Several aspects of the primary tumor microenvironment are known to play a key role in aiding metastases to the LN [16,17,18]. Increased expression of vascular endothelial growth factors C and D (VEGFC and VEGFD) in the primary tumor is known to increase the dissemination of tumor cells to the lymph nodes, primarily through lymphangiogenesis, a process by which existing lymphatic vessels migrate towards the primary tumor [19,20,21,22,23]. Antagonists against VEGF have been shown to effectively inhibit LN metastases [24,25,26]. The mechanism by which cancer cells enter lymphatic vessels is poorly understood (Figure 1). However, it has been hypothesized that the lack of pericytes and basement membrane in lymphatic blood vessels ease the lymphatic spread of cancer [27]. To reach the lymphatic vessels, cancer cells must sense directional cues in the tumor microenvironment. Several chemokines expressed by the endothelial cells lining the lymphatic vessels and their receptors expressed by cancer cells have been demonstrated to play a significant role in the lymphatic spread of cancer [28,29]. Chemokine receptors CXCR4 and CCR7 and their respective ligands CXCL12 and CCL21 play an important role in the migration of breast cancer cells to the lymphatic vessels [30]. CCR7 and CCR10 and their respective ligands CCL21 and CCL27 have been implicated in the migration of melanoma cells to the lymphatic vessels [31]. Unlike the circulatory system, the lymphatic system does not have a central pump to regulate the flow of lymph [32], resulting in relatively low flow velocities. The flow of lymph is guided by a pressure gradient in lymphatic capillaries [33]. The high interstitial pressure within the tumor has been proposed as a driving mechanism for the movement of tumor cells through the lymphatic drainage pathway [34,35]. Thus, cancer cells can be passively translocated to the TDLN once they are inside the vessel lumen. Bloodborne cancer cells can also reach the TDLN by mimicking leukocyte trafficking patterns. LN have specialized postcapillary venules called high endothelial venules (HEV) that enable circulating lymphocytes in the blood to directly enter LN [36,37]. The trafficking of intravascular lymphocytes to the LN is mediated by rolling, adhesion and transmigration of lymphocytes through the endothelial cells lining the HEV [13]. The adhesive interactions between cancer cells and endothelial cells are considered to be important in the metastatic cascade [38]. For instance, in the metastases of hematologic cancers such as leukemia and lymphoma to the TDLN, these adhesive interactions are mediated by L-selectin expressed on cancer cells and peripheral node addressin (PNAd) expressed on endothelial cells in the HEV [39,40]. In other types of cancers, these interactions may be mediated by E-selectin expressed on endothelial cells in the HEV and E-selectin ligands expressed on cancer cells [38].

Cancer cells in the TDLN often form micrometastatic lesions that can remain dormant for several years before becoming overt metastases [41]. Micrometastases are small aggregates (<2 mm) of cancer cells that are clinically undetectable using conventional histopathology [42]. This has led to the development of new assays based on immunohistochemistry using a distinct set of markers [43,44,45] or assays based on polymerase chain reaction [46,47] to estimate the prevalence of micrometastases in cancer patients. Metastases in the TDLN eventually lead to distant organ metastases but the underlying mechanisms are poorly understood. Current understanding of the role of the TDLN in orchestrating distant organ metastases is a “black box” [48] because the intermediary steps are not distinctly clear. The presence of immune cells that can elicit an anti-tumor response does not prevent cancer cells from metastasizing to the TDLN. The prospective of employing the immune system to fight cancer is very appealing and has led to the development of a class of therapeutic approaches often termed “tumor immunotherapy” [49]. The TDLN have a unique position as the microenvironment can induce an anti-tumor response and at the same time can be involved in the malignant spread of cancer [50,51,52]. The potential for targeted immunotherapy to the TDLN has been researched extensively [53,54]. The proximity of the TDLN to the primary tumor and the free passive flow of lymph from the primary tumor to the TDLN prove advantageous for targeted therapies to the TDLN. This review will focus on the microenvironment of the TDLN and then discuss liposome-based approaches to induce an anti-tumor immune response in the TDLN.

## 2. Microenvironment of the Tumor-Draining Lymph Node

**Figure 2:**
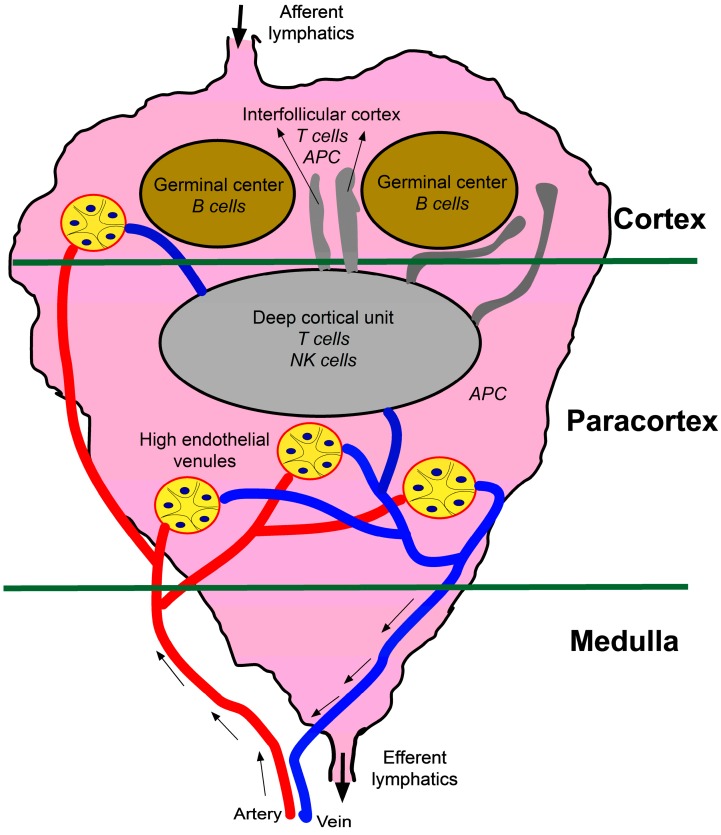
Schematic of a lymphoid lobule: The structural and functional unit of a LN is a lymphoid lobule. The number of lymphoid lobules per LN can vary depending on the size of the LN. The lymph enters the LN through the afferent lymphatics, is filtered through the LN and exits through the efferent lymphatics. The immune cells reach the LN through the high endothelial venules and are compartmentalized within the lymphoid lobule. The lymphoid lobule can be divided into three regions: cortex, paracortex and medulla. The cortex has germinal centers with B cells and an interfollicular cortex containing T cells and antigen presenting cells (APC). The paracortex is mostly filled with APC and has the deep cortical unit containing T-cells and NK cells. The medulla mostly consists of veins and lymphatic capillaries that drain the LN.

The structural and functional unit of the LN is a lymphoid lobule (Figure 2), and the number of lymphoid lobules can vary from a few to several thousand depending on the size of the LN [9]. Lymph fluid containing the local immune signature enters the LN through afferent lymphatic vessels and is channeled around the lymph node through conduit channels [55]. These channels are lined with macrophages and DC that can remove microorganisms and antigens from the lymph and present them to T- and B-cells [56]. Lymphocytes home to the LN from blood through the HEV. T- and B-cells are compartmentalized within the lymphoid lobule, where they interact with APC and undergo expansion. The cortex contains germinal centers (B-cell area) and interfollicular cortex (T-cells). The paracortex consists of a deep cortical unit containing T-cells. APC tend to be in the paracortex and interfollicular cortex of the LN. The medulla is largely composed of channels and blood vessels that drain the LN.

**Figure 3:**
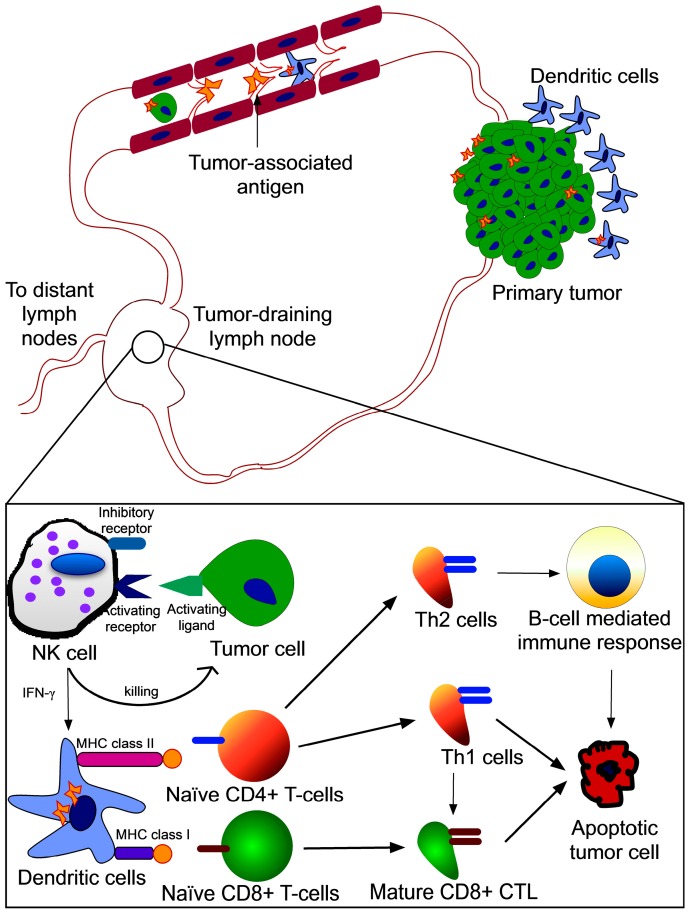
Immune responses in the TDLN: Tumor-associated antigens (TAA) can elicit an anti-tumor immune response in the TDLN through three possible routes: (1) DC in the TDLN ingest TAA transported via passive flow through the lymphatic capillaries to activate naïve T-cells by cross-presentation of antigens; (2) Tumor-infiltrating DC internalize TAA at the primary site and are then transported to the TDLN to activate naïve T-cells; (3) Tumor cells in the TDLN directly present the antigens to naïve T-cells. Once cancer cells reach the TDLN, NK cells are activated that kill tumor cells using effector molecules such as TRAIL and perforins. Activated NK cells secrete immune stimulatory cytokines such as IFN-γ that further activate DC. TAA are presented to naïve T-cells in the TDLN. Antigen presentation on MHC class II molecules activates naïve CD4+ T-cells that differentiate into T-helper (Th) cells. Th2 cells induce cancer cell death via B-cells and Th1 cells directly mediate cancer cell death. Antigen presentation on MHC class I molecules activates naïve CD8+ T-cells that differentiate into cytotoxic T-cells (CTLs) to induce cancer cell death.

The immune system has potential for keeping tumor growth under control. The main function of the immune system is to maintain tissue homeostasis by protecting against infectious pathogens/allergens and eliminating damaged cells. Immunologic tolerance prevents an immune response against self-antigens. When tissue homeostasis is chronically perturbed, as in cancer, complex and paradoxical interactions between immune cells and cancer cells can occur that lead to a breakdown of self-tolerance. Leukocytes in and around the developing tumor have been associated with reducing tumor burden [57,58,59]. The adaptive immune response to tumors is directed against tumor-associated antigens (TAA) expressed specifically by tumor cells [60]. The role of TAA-specific T-cell response has been extensively characterized [61]. There is experimental evidence from mouse models of cancer demonstrating the roles of both adaptive and innate immune responses in controlling tumor progression [62,63]. See Ref. [64] for a detailed review on tumor immune surveillance. Tumor progression is a balance between anti-tumor response by the host immune system and immune suppression by tumor cells. Most of the immune surveillance takes place in the TDLN due to its cellular composition and proximity to the primary tumor. TDLN, although very small in size, can have a profound influence on anti-tumor immune response since it is the hub of immune surveillance. Unfortunately, the microenvironment of the TDLN is immune-suppressed in cancer patients, thereby favoring tumor progression and eventually affecting systemic immune response against tumor cells. For a detailed understanding of the molecular basis of immuno-evasion by tumor cells, readers are referred to excellent reviews by Dunn *et al*. [65,66]. Here we summarize the anti-tumor immune response mediated by different types of cells in the TDLN (Figure 3) and how they are affected by the immunosuppressive microenvironment of the TDLN.

### 2.1. Natural Killer Cells

NK cells are the first type of innate immune cell to participate in host immune surveillance because of their ability to initiate an anti-tumor response without being able to recognize a TAA [67]. They represent 5%–15% of circulating blood lymphocytes [68] and play a key role in the host antimicrobial and antiviral response [69]. NK cell activity is a delicate balance between activating and inhibitory receptors expressed on the surface of NK cells [70]. They can be activated by cells that lack the expression of MHC class I molecule and induce a cytolytic response predominantly mediated by interferon-γ (IFN-γ) and tumor necrosis factor-α related apoptosis-inducing ligand (TRAIL) [71]. Total or partial loss of MHC class I molecule has been reported in several types of human tumors [72,73]. Experimental studies in mice show that NK cells are involved in the eradication of tumors. Mice depleted of NK cells have shown uncontrollable tumor growth when compared to wild type mice [74,75]. NK cells are derived from pluripotent stem cells in the bone marrow [76]. Precursor NK cells migrate to the LN through the HEV and the local chemokine environment plays an important role in their maturation process [77]. However, NK cells in cancer patients have several abnormalities, including reduced count, decreased cytotoxicity and poor tumor infiltrating capacity [78] owing to the immunosuppressive microenvironment in cancer patients. Several studies show that LN contain mature NK cells that can elicit an anti-tumor immune response [79,80,81,82]. IFN-γ secreting NK cells have been identified in the paracortex of LN using two-photon microscopy [83]. Mature cytotoxic NK cells were identified to infiltrate the TDLN in patients with metastatic melanoma [84]. The expression of activating receptors and effector molecules on NK cells derived from the TDLN of patients inversely correlated with the percentage of tumor cells, indicating local immune suppression of NK cells by melanoma cells [84]. However, several studies indicate the inability of NK cells in the TDLN to elicit anti-tumor immune response [85,86,87,88]. For instance, the tumoricidal activity of NK cells isolated from the TDLN of patients with head and neck carcinomas was significantly suppressed [85]. NK cells isolated from invasive breast cancer patients showed altered phenotype and function that allowed cancer cells to escape NK-cell mediated anti-tumor response [86]. Also, cancer cells can escape NK-cell mediated immune response by upregulating MHC class I analogues [87] or downregulating death receptors that mediate NK-cell mediated apoptosis of cancer cells [88]. Thus, despite their presence in the TDLN, NK cells may lack the ability to eliminate cancer cells.

### 2.2. T-Cells

T-cells are the most important cells of the adaptive immune response. T-cells express the T-cell receptor (TCR) that can recognize TAA on MHC molecules on tumor cells and APC [89,90,91]. The TAA can be directly presented on MHC class I molecule on tumor cells [92] or presented on either MHC class I or class II molecules on DC [93]. T-cell precursors from the bone marrow become naïve T-cells in the thymus and reach the LN through HEV. There are two major subtypes of T-cells based on the expression of two different coreceptors. T-cells that co-express CD8 recognize peptides presented on MHC class I molecules expressed predominantly by all nucleated somatic cells. CD4+ T-cells recognize TAA presented on MHC class II molecules expressed specifically by APC [94,95]. CD8+ T-cells are cytotoxic T-cells that recognize TAA presented on MHC class I molecules and release perforins and other effector molecules to induce tumor cell death [96]. CD4+ T-cells are further categorized into T-helper (Th) cells or T-regulatory (T-regs) cells. T-helper cells mediate cellular and humoral response whereas T-regulatory cells suppress self-reactive T-cells. TAA can be presented on MHC class I molecules in the TDLN, in which case CD8+ cytotoxic T-cells are activated that lyse the cancer cells. TAA can also be presented on MHC class II molecules, in which case CD4+ T-helper cells are activated that mediate the cellular or humoral immune responses. T-cells are mostly restricted to the deep cortical unit and the interfollicular cortex within the LN. The TDLN have fewer HEV than normal LN, resulting in fewer naïve lymphocytes that can mediate anti-tumor response [51]. There is also significant reduction in T-cell recruitment through the HEV leading to a decrease in anti-tumor response in the TDLN [51]. Functional T-cells are formed when APC present antigens to naïve T-cells. The differentiation of naïve T-cells is mediated by immune stimulatory cytokines secreted by cells in the LN. Characterization of cytokine profiles of the TDLN in patients with gastrointestinal cancer have revealed significantly lower levels of cytokines that mediate cellular immunity than LN in healthy individuals [97]. This leads to fewer mature T-cells in the TDLN than LN from healthy individuals. The local cytokine environment in the TDLN has been shown to be immune-suppressive in melanoma patients [98]. The tolerogenic behavior of T-cells in the TDLN is due to increased activity of immunosuppressive T-regs in the TDLN [99,100,101,102,103,104,105]. The depletion of T-regs in the TDLN has improved anti-tumor immune response in mice [106]. The presence of T-regs has also been shown to interfere with the proliferation of adoptively transferred CD8+ cytotoxic T-cells [107]. Tumor progression in mice has been associated with an increase in the number of T-regs in the TDLN [108]. T-regs secrete immunosuppressive cytokines such as interleukin-10 (IL-10) and transforming growth factor-β1 (TGF-β1) that interfere with the activity of CD8+ cytotoxic T-cells [109]. The anti-tumor response mediated by T-cells in the TDLN is dampened by an increase in the number of T-regs that secrete immunosuppressive cytokines, which hinder the differentiation of naïve T-cells when they are presented with antigens by APC.

### 2.3. Antigen Presenting Cells

APC may not be the most predominant type of cells in the TDLN, but they play a central role in tumor immunosurveillance. A particular type of APC, the DC, initiates anti-tumor response in the TDLN. DC are known to infiltrate solid tumors in tumor-bearing mice and patients [110]. Efficient priming of T-cells against tumors is dependent on TAA presentation by DC. TAA are soluble proteins secreted by tumor cells or encapsulated in exosomes derived from tumor cells [111]. TAA initiate anti-tumor responses through tumor-infiltrating DC at the primary site or through DC that reside in the TDLN [112]. DC at the primary site ingest TAA and migrate to the TDLN to activate naAA aT-cells, or free TAA in the lymph reach the TDLN to activate DCs in the TDLN. DC capture and internalize TAA through a number of receptors, including Fc receptors, CD11c/CD18, DEC205 and Toll-like receptors [113]. The uptake of TAA by DC initiates a cascade of events that triggers their maturation process. It has been shown that tumor-infiltrating DC and those found in the TDLN share an immature phenotype [114,115]. The stimulation of T-cells by DC is dependent on antigen recognition by T-cell receptor (TCR) and co-stimulatory signal from DC that leads to T-cell proliferation. DC are known to express different types of T-cell co-stimulatory molecules such as B7 [116], CD40 [117], 4-1BB [118] and ICAM-1 [119]. The microenvironment of the TDLN could prevent the efficient uptake and presentation of TAA by DC (tolerance), hinder their ability to co-stimulate T-cells (anergy) or activate immune-suppressive T-regs cells (suppression). Studies in mice have shown that DC in the TDLN are very poor stimulators of T-cells [120]. In mice, the TDLN house DC that express the immunosuppressive enzyme indoleamine 2,3-dioxygenase (IDO) [121,122]. DC expressing IDO are known to suppress the T-cell mediated anti-tumor response by activating T-regs [121,122]. There are several factors that contribute to dysfunctional DC in the TDLN. For a detailed review on the behavior of dendritic cells in the TDLN, readers are referred to Ref. [123].

### 2.4. B-Cells

T-helper cells activate B-cells that mediate the humoral immune response [124]. B-cells can be categorized into two major subtypes: plasma cells and memory cells. Plasma cells secrete antibodies against TAA that mark tumor cells for T-cell mediated immune response [124,125]. Memory cells elicit an anti-tumor response at a systemic level. It has been shown that 30%–35% of cells of the TDLN in mice are comprised of B-cells [126]. Unlike T-cells, the role of B-cells in the TDLN has not been widely studied. B-cells can also function as APC to T-cells [127]. It was recently shown that B-cells in tumor draining lymph nodes from melanoma patients are responsible for activating a CD4+ T-cell mediated anti-tumor response [128]. The TDLN in cancer patients are also known to show an up-regulation of a subset of B-cells called regulatory B-cells that secrete immunosuppressive cytokines such as IL-4, IL-10 and TGF-β that curb T-cell mediated immune response [129,130].

### 2.5. From Local Tolerance to Systemic Tolerance

The microenvironment of the TDLN may have a profound influence on anti-tumor immune response and distant organ metastases. Normally, LN favor immune activation against a pathogen or TAA owing to the presence of cells that constitute the central part of the adaptive and innate immune responses. However, in cancer patients, the TDLN under the influence of a tumor becomes a site of immune suppression. The tolerogenic phenotype of cells in the TDLN stems from the ability of cancer cells to secrete chemokine factors that can alter the microenvironment of the TDLN. The pressure gradient from the primary tumor to the TDLN passively transports TAA, cytokine and chemokine factors from the primary tumor microenvironment. The cells in the TDLN can further amplify the tolerogenic milieu. For a detailed review of immunosuppressive cytokines secreted by tumor cells and their role in immune evasion, see Ref. [131,132]. The immunosuppressive phenotype of the TDLN is a combinatory effect of pre-metastatic cytokines secreted by the primary tumor and post-metastatic cytokines secreted by cancer cells in the TDLN. Experimental preclinical study in mice bearing human melanoma cells indicate that immunomodulatory cytokines secreted in primary tumor-derived exosomes can condition the TDLN and suppress the anti-tumor immune response to prepare them for metastasis [133]. Characterizing the immunosuppressive phenotype of lymph nodes in melanoma patients indicate that the immunosuppression precedes lymph node metastasis, showing that cytokines secreted by a primary tumor can modulate the microenvironment of the TDLN [134]. Alternatively, comparing the gene expression pattern on breast cancer patient lymph nodes with and without metastatic lesions in their lymph nodes indicate a significant down-regulation of key immunosuppressive genes in patients with lymph node metastasis, suggestive of a post-metastatic effect of cancer cells in the immunosuppressive phenotype of the TDLN [135]. The local tolerance can have an effect on the entire immune system because the average number of LN in an adult human is about 450 and they are connected by a vast network of lymphatic capillaries [9]. Systemic tolerance can be induced by either anergic T-cells or activated T-regs that migrate through the lymphatic capillaries. When a tumor cell metastasizes to a different tissue, the LN of that tissue is presented with TAA and this triggers an anti-tumor immune response. The systemic tolerance can affect the ability of immune cells in LN draining the metastatic site to act against TAA, thereby favoring distant organ metastasis. Cancer cells may also translocate through the immune tolerant lymphatic system, offering a route for distant organ metastases. The communication between the lymphatic system and the circulatory system provides an opportunity for T-regs and tolerogenic immune cells to enter the peripheral circulation, thus posing a greater threat of metastatic disease.

## 3. Liposome-Based TDLN Targeted Immunotherapy

Cancer immunotherapy relies on the stimulation of the host immune system to attack tumor cells. Several strategies have been proposed to circumvent the immunosuppressive microenvironment of the TDLN. Previous studies have shown that activating DC in the TDLN using TAA and cytokines can overcome T-cell anergy [136,137,138,139,140,141]. Clinical trials for cancer treatment in human subjects do not follow the same trend as preclinical studies in mice [142]. This is because of the dosage of immune stimulating agents used in preclinical studies could be toxic in humans and is often scaled down in clinical trials involving human subjects. For instance, IL-2 therapy is widely used in the treatment of metastatic melanoma [143,144]. The systemic activation of the immune system using IL-2 causes severe side effects in cancer patients [145]. To circumvent the side effects of systemic activation, several alternative therapies are being developed to activate the host immune system. Cell-based immunotherapeutic approaches involve autologous infusion of isolated immune cells or cancer cells (manipulated *ex vivo*) from patients [146]. Cancer cells can be manipulated to express more TAA [147] or T-cell co-stimulatory molecules [148]. Most of the immune cell-based approaches involve *ex vivo* manipulation of DC [149] and T-cells [150]. The disadvantage of DC cell-based immunotherapeutic approach is that mature DC do not express L-selectin, which mediates the translocation of infused DC from the systemic circulation into the TDLN [13].

**Figure 4:**
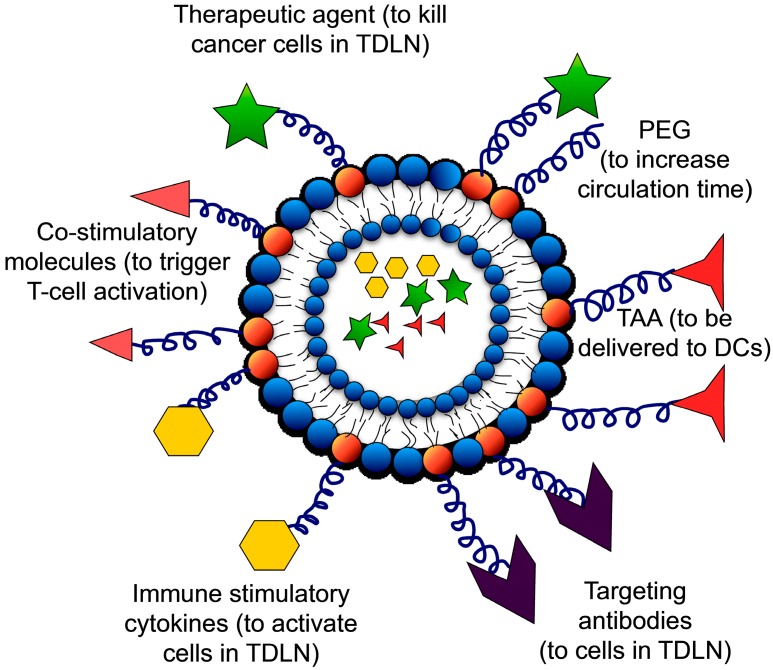
Schematic of liposomes used in TDLN-targeted immunotherapy: Liposomes are composed of lipids with polyethylene glycol (PEG) to increase their circulation time. They can be encapsulated with TAA, immune stimulatory cytokines and therapeutic agents to kill cancer cells. They are functionalized with proteins either by using chelator lipids (his-tagged proteins) or using PEG with maleimide head groups (thiolated proteins). They can be functionalized with immune stimulatory cytokines (e.g., IL-2), co-stimulatory molecules (e.g., anti CD-40 and anti CD-137), therapeutic agents (e.g., TRAIL), targeting antibodies (e.g., anti-DEC205, anti-CD11c to DC and anti-CD57 to NK cells) and TAA (e.g., ovalbumin).

The key players of anti-tumor immunity are present in the TDLN. To generate immunity against tumor cells, therapies have to be directed towards the TDLN. Nanoscale targeted therapies that prime the adaptive immune system have been successful in generating an effective response against tumor cells. Most of the targeted therapies are directed towards DC and T-cells in the TDLN because they play a key role in inducing the cellular and humoral immune responses. Nanoscale bioengineering techniques apply engineering approaches to address problems in drug delivery, synthetic implants and tissue engineering. Several nanomaterial-based approaches have been proposed to deliver antigens and adjuvants to trigger the host immune system [151]. Liposomes are small nanoscale vesicles that are produced by suspending natural and synthetic lipids in aqueous buffer [152]. The discovery of stealth-liposomes by conjugating polyethylene glycol (PEG) on the lipid head groups is a major advancement in liposome-based targeted drug delivery approaches [153]. They have a longer lifetime in blood owing to their increased stability and decreased interaction with blood components. Liposomes used in the TDLN-directed immunotherapy (Figure 4) are structures largely composed of natural and synthetic phospholipids that are encapsulated with TAA or immune stimulatory cytokines and functionalized with recombinant cytokines/co-stimulatory proteins that activate immune cells. They are also functionalized with proteins that target them to a specific cell type in the TDLN. They are also encapsulated and/or functionalized with therapeutic drugs that can kill cancer cells. Liposomes are a good alternative to systemic and cell-based immunotherapeutic approaches because of their ability to specifically target TDLN and activate long-term anti-tumor immune response without detrimental side effects.

### 3.1. Factors Affecting the Delivery of Liposomes to Lymph Nodes

Liposome size, surface charge, lipid composition, PEG chain length and site of injection can affect the delivery of liposomes to the TDLN [154]. Liposomes have an advantage for delivering therapeutic substances to the LN because of their size. Typically liposomes are ~100 nm in size, which is often too large to be directly absorbed into the peripheral circulation but small enough to enter the lymphatic circulation following different modes of administration such as subcutaneous, intra-muscular, or direct injection into organs or tumors [155]. The mode of injection and the type of targeting moiety on the liposome surface are two major factors that determine the effective delivery of liposomes to LN [156]. For a detailed understanding of factors influencing lymphatic absorption and lymph node uptake of liposomes, readers are referred to Refs. [154,155]. Subcutaneous and intra-tumoral delivery have been widely used in TDLN-directed liposome-based preclinical studies. A targeting agent can be functionalized on the surface of liposomes using maleimide-thiol chemistry [157] or by including a chelator lipid in the original liposome composition that can bind to his-tagged proteins [158]. Facilitated delivery without any targeting molecules has also been exploited because of the ability of liposomes to passively reach the TDLN when injected directly into the tumor. Liposomes have been shown to interact with monocytes and DC without any targeting molecule [159]. Cell-derived plasma membrane vesicles (PMV) are widely used in TDLN-directed therapies. PMV can be isolated by sonication of cells and high-speed centrifugation in sucrose gradient. PMVs can be modified like liposomes to encapsulate antigens and functionalized with antibodies to target them to cells in the TDLN [156].

### 3.2. Delivering Liposomes to the TDLN by Targeting DC

DC are initiators of adaptive immunity and are often exploited for liposome-based targeted therapies to the TDLN. Nanoscale liposomes, which are several orders of magnitude smaller than DC, can deliver TAA to DC to promote antigen-specific T-cell response [53,113]. Encapsulating or functionalizing TAA on the liposome surface has been extensively studied for antigen delivery to DC. It has been shown that functionalizing TAA encapsulated liposomes or plasma membrane vesicles (PMV) with antibodies that recognize DC can elicit a strong anti-tumor response in mice [160]. The most widely studied model is ovalbumin (a model antigen) encapsulated liposomes targeted to murine DC. DC process ovalbumin and trigger ovalbumin specific cytotoxic T-cells. Mice are often challenged with cancer cells (transfected with plasmids to express ovalbumin) to evaluate the efficacy of this approach. The receptors CD11c/CD17 and DEC-205 are expressed on DC and they play important roles in antigen presentation. Liposomes and PMV functionalized with single chain variable fragments (ScFv) derived from anti-CD11c and anti-DEC-205 are able target murine DC upon subcutaneous injection [113]. Encapsulating engrafted liposomes and PMVs with ovalbumin and immune stimulatory cytokines such as IFN-γ drastically reduced the number of lung metastases in mice injected with B16-OVA cells. The absence of immune stimulatory cytokines or DC targeting antibodies did not have any effect on the number of lung metastases observed. DC also express mannose receptor (CD206) to recognize pathogens and this has been exploited for delivering liposomes targeted to DC [161]. Oligomannose-coated liposomes (OML) have been used to deliver tumor antigens to dendritic cells [162,163,164,165] by interacting with CD206. Upon subcutaneous injection in mice, fluorescent BSA containing OML accumulated in the draining LN [164]. Mice were immunized with OML containing ovalbumin (OML/OVA) or bare liposomes (bare/OVA) with ovalbumin and challenged with E.G7-OVA tumor cells. Mice immunized with OML/OVA were able to completely reject E.G7-OVA tumor cells and harbored cytotoxic T-cells that were able to secrete significantly higher levels of immunosuppressive cytokines such as IL-4 and IFN-γ. OML/OVA treatment also had an effect on established E.G7-OVA tumors. Mice were inoculated with E.G7-OVA tumor cells and after the tumor growth became palpable, they received subcutaneous injections of OML/OVA, bare/OVA or PBS. Significant suppression of tumor growth was observed in mice injected with OML/OVA.

Liposomes functionalized with immunomodulatory agents that stimulate DC have also been used in TDLN-directed immunotherapy. CD40 is a receptor expressed on DC that recognizes CD40 ligand expressed on T-cells and is involved in co-stimulation of T-cells [166]. Anti-CD40 is an extensively used immune-stimulatory agent that can bind to CD40 on DC and induce a potent T-cell mediated immune response [167,168]. Systemic injection of anti-CD40 can produce detrimental side effects in cancer patients and liposome based delivery of anti-CD40 to the TDLN has been shown to reduce harmful side effects often observed in systemic treatment [169]. CpG oligonucleotide is a ligand for Toll-Like Receptor 9 (TLR9) expressed on DC. Stimulation of TLR9 by CpG oligonucleotide results in the stimulation of T-cells that secrete immune-stimulatory cytokines to promote tumor regression [170,171]. Similar to anti-CD40 systemic treatment, CpG can produce dangerous side effects [172]. Liposomes were functionalized with CpG oligonucleotides using CpG–DNA lipid conjugates. Liposomes were also functionalized with anti-CD40 using maleimide-thiol conjugation to study the combinatory effect of anti-CD40 and CpG [173]. The anti-CD40/CpG liposomes exhibited controlled release of anti-CD40 and CpG over a period of seven days, thereby reducing the risk of systemic toxicity. Mice were inoculated with B16F10 tumor cells, which were allowed to establish for eight days. Tumor-bearing mice were given intra-tumoral injections of PBS (control), soluble anti-CD40 alone, soluble CpG + anti-CD40 and anti-CD40/CpG liposomes. The combination liposomes were able to delay but not prevent tumor progression. This study is an example of how intra-tumoral injection of therapeutic substance can help in targeting the TDLN. Flow cytometry analysis of surface CpG expression in CD11c positive DC in the TDLN after intra-tumoral injection revealed that the TDLN took up liposomes, indicating successful delivery of therapeutic liposomes.

The use of pH-sensitive polymers has also been extensively used for inducing antigen-specific immunity [174]. Modifying the surface of liposomes with pH-sensitive polymers has been applied to deliver tumor antigens into the cytosol of DC [175,176]. Delivery of antigens into the cytosol of DC is critical for antigen presentation and subsequent activation of T-cells. pH-sensitive polymers are stable at neutral pH, but become destabilized under acidic pH conditions of the endosomes, releasing their contents into the cytosol. For more discussion on applications of pH-sensitive liposomes, see Ref. [174]. Recently, two types of pH-sensitive poly(glycidol) derivatives with either a linear (MGlu-LPG) or hyperbranched (MGlu-HPG) structure were developed to study the delivery of antigen into DC [175]. FITC-OVA loaded fluorescent pH-sensitive liposomes were added to isolated DC and incubated for 4 h at 37 °C. Confocal laser microscopy revealed that pH-sensitive polymer-modified liposomes were able to successfully deliver fluorescently tagged antigen (FITC-OVA) to the cytoplasm of DC *in vitro*. pH-sensitive liposomes encapsulated with antigen increased the expression of MHC class I and II molecules when incubated with DC *in vitro*, suggesting their ability to activate DC. Mice were immunized with OVA solution, OVA loaded unmodified liposomes or OVA-loaded pH-sensitive liposomes (MGlu–LPG or MGlu–HPG) by receiving subcutaneous injections. One week later, E.G7-OVA cells were inoculated subcutaneously into the backs of mice. MGlu–HPG-modified OVA-loaded liposomes were able to completely reject the tumor cells inoculated into the mice, showing that pH-sensitive liposomes had the greatest ability to induce an antigen-specific immune response. To evaluate the therapeutic effect of polymer-modified liposomes, mice were first inoculated with E.G7-OVA cells and one week after inoculation; modified and unmodified liposomes loaded with OVA were subcutaneously administered into tumor bearing mice. Tumor volume was significantly decreased for mice treated with MGlu–HPG-modified OVA-loaded liposomes. More extensive shrinkage of tumor burden was seen in mice immunized with MGlu–LPG-modified OVA-loaded liposomes. These results show that pH-sensitive polymer-modified liposomes are quite effective in inducing an anti-tumor immune response. A more biocompatible alternative to the same approach was recently developed by using dextran instead of poly(glycidol) derivatives (MGlu–Dex liposomes) [176]. For clinical trials, the pH-sensitive polymers must be biologically safe and biodegradable, and dextran as a naturally occurring polysaccharide satisfies this requirement. The MGlu–Dex modified liposomes were also able to successfully deliver antigens to DC and induce a potent anti-tumor immune response [176].

### 3.3. Delivering Liposomes to the TDLN by Targeting T-Cells

The immune-suppressive microenvironment of the TDLN has anergic T-cells because of the inability of DC to provide co-stimulatory molecules [138]. In the absence of appropriate co-stimulatory signals delivered through T-cell surface molecules such as CD28, CD40L and 4-1BB, the recognition of antigens on MHC class I molecules by TCR leads to the accumulation of unresponsive T-cells [177,178]. Though liposomes targeted to DC results in successful antigen presentation, induction of T-cell anergy often enables the tumor cells to escape the immune response in the TDLN. Thus, liposomes functionalized with co-stimulatory signals for T-cells such as 4-1BBL and immune stimulatory cytokines such as IL-2 have been used to prime T-cells to induce an anti-tumor response [179]. CD137 (4-1BB) is a co-stimulatory receptor expressed by T-cells that binds to CD137L (4-1BBL) expressed by activated DC [118]. External stimulation of CD137 using antibodies has been shown to induce an anti-tumor immune response in several studies [180,181]. IL-2 is an immune stimulatory cytokine that activates T-cells by binding to its receptor and has been approved for clinical immunotherapy against metastatic melanoma [182]. IL-2 treatment using intravenous injection is only partially successful and produces severe side effects [183,184]. IL-2 functionalized liposomes directed towards adoptively transferred T-cells with antibody recognizing T-cells were shown to induce T-cell proliferation *in vivo* [185]. Liposomes were functionalized with antibody against Thy1.1 (CD90) to specifically target T-cells and recombinant IL-2 to induce T-cell proliferation [185]. Given the ability of antibody-functionalized liposomes to target adoptively transferred T-cells *in vivo*, studies were directed towards targeting T-cells in the TDLN. Using a murine model, it was shown that localized TDLN targeted therapy using liposomes functionalized with anti-CD137 and IL-2 can control tumor progression by activating a systemic anti-tumor response [179]. Liposomes were able to bind to murine T-cells *in vitro* and induce the proliferation of T-cells. Flow cytometric analysis of T-cells isolated from the TDLN and spleen of mice that received intra-tumoral injection of fluorescent liposomes confirmed the lymphatic drainage of liposomes and their ability to bind to T-cells in the TDLN. Mice were inoculated with B16F10 cells and after 10 days received intra-tumoral injections (three doses over the course of one week) of anti-CD137 and IL-2 either conjugated to liposomes or in soluble form. Tumor growth was monitored for up to 60 days and tumor regression was observed in 60%–70% of mice treated with soluble therapy or liposome-based immunotherapy. However, combinatory liposome therapy was able to induce an anti-tumor immune response without side effects, such as dramatic loss of body weight or elevated levels of potenially toxic inflammatory cytokines. The localized immunotherapy also had an effect on systemic immunity by inhibiting metastasis of tumors to distant sites. Mice received subcutaneous injections of B16F10 tumor cells simultaneously in both flanks but received intra-tumoral injections of combinatory liposomes on just one flank. The average tumor growth was monitored on both flanks. The treated tumors were either completely eliminated or showed significantly delayed progression while tumor growth on the other flank was also significantly inhibited. This indicates that localized immunotherapy directed towards the TDLN can also have a beneficial effect on systemic anti-tumor response. T-cells were isolated from the TDLN and distal LN of tumor bearing mice and the number of T-cells were enumerated with or without *ex vivo* stimulation. The density of IFN-γ secreting CD8+ and CD4+ T-cells was increased in both the TDLN and distal lymph nodes in treated mice. It was shown that localized immunotherapy activated T-cells in the TDLN, which then disseminated to distant sites to promote tumor regression. This indicates that immunotherapy directed towards the TDLN can affect how the immune system reacts to tumor cells throughout the body and not just at the site of injection. While intravenously injected soluble cytokines and agonists can also affect how the immune system responds to tumors, it has the considerable disadvantage of elevating the serum levels of immunomodulatory cytokines to toxic levels. Attaching these immunomodulatory cytokines to liposomes provides a platform for controlled release and targeted delivery to the TDLN that can lead to systemic anti-tumor immune response without detrimental side effects.

### 3.4. Delivering Therapeutic Liposomes to TDLN by Targeting NK Cells

**Figure 5:**
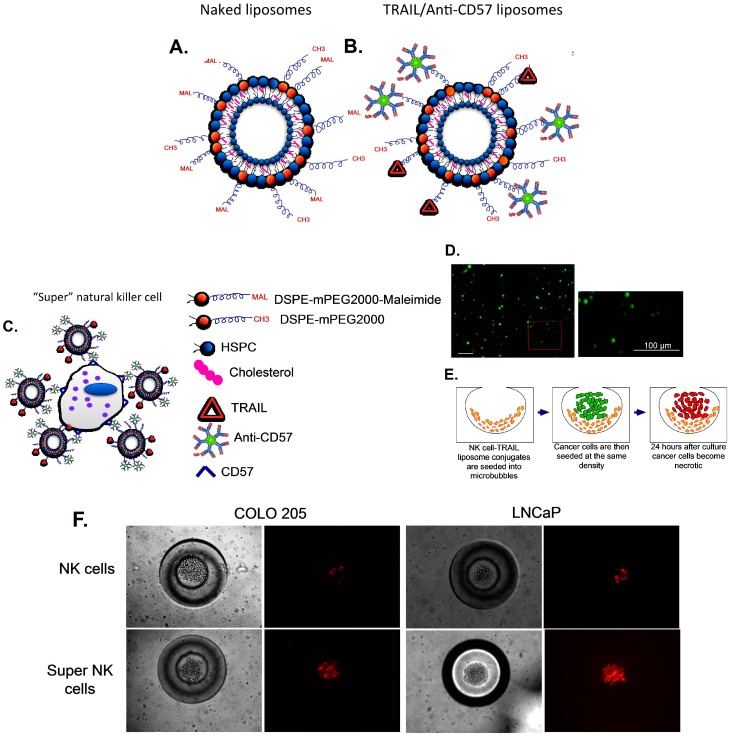
Delivering therapeutic TRAIL-liposomes to the TDLN by targeting NK cells: (**A**) Schematic of liposomes with maleimide functional group; (**B**) thiolated anti-CD57 and TRAIL covalently bound to liposomes; (**C**) “Super” NK cells are liposome functionalized NK cells via antibody binding to CD57 on the surface of NK cells; (**D**) confocal micrographs of NK cells conjugated to fluorescent liposomes with inset showing an even distribution of liposomes on the NK cell surface (Scale bar = 100 μm) (**E**) “Super” NK cells and LN-seeking cancer cells cultured in 3D microbubbles; and (**F**) Fluorescent micrographs of COLO205 and LNCaP cells cultured with endogenous NK and “super” NK cells for 24 h in microbubbles incubated with propidium iodide (red) to stain for necrotic cancer cells. Reproduced by permission of the Royal Society of Chemistry from Ref. [187]. DSPE: 1,2-distearoyl-*sn*-glycero-3-phosphoethanolamine; HSPC: hydrogenated soy phosphatidylcholine; PEG: polyethylene glycol.

Cancer cells in the TDLN escape NK-cell mediated immune response by expressing MHC class I like analogues, secreting soluble ligands that can bind to NK cell receptors or by reducing the expression of effector ligands such as TRAIL on NK cells [186]. NK cells are the most aggressive subset of T-cells but NK cells in the TDLN of cancer patients have several abnormalities, ranging from reduced count, poor cytotoxicity and weak tumor infiltrating capacity. Thus, to target cancer cells in the TDLN, we recently proposed a liposome-based targeted drug delivery approach to enhance the efficacy of endogenous NK cells [187]. TRAIL is an important effector molecule expressed by NK cells and is known to play an important role in NK-cell mediated anti-tumor response [71]. TRAIL is a type II transmembrane heterodimer with a molecular weight of 60 kDa with a half-life of 5–8 min in blood and is rapidly eliminated from the serum primarily through the kidneys [188]. By functionalizing TRAIL-liposomes with an antibody against NK cells (anti-CD57), we hypothesize that NK cells can serve as a carrier for TRAIL functionalized liposomes (becoming “super” NK cells). In an attempt to enhance the therapeutic potential of NK cells to overcome immune suppression in the TDLN, the surface of NK cells was functionalized with TRAIL liposomes to kill cancer cells. Since NK cells are found in the paracortex of LN, liposomes functionalized with NK cell directed antibody and TRAIL could specifically target cancer cells in the TDLN. Thiolated TRAIL and anti-CD57 proteins were covalently bound to the maleimide functional groups on the surface of liposomes (Figure 5A,B). Conjugation of TRAIL-functionalized liposomes to NK cells is mediated by CD57 expressed on the surface of NK cells and anti-CD57 conjugated to the liposome surface (Figure 5C,D). NK cells were isolated from whole blood and incubated with TRAIL/anti-CD57 liposomes to form “super” NK cells. Microfabrication has led to the development of 3D cell culture platforms using biocompatible materials to mimic *in vivo* microenvironment. We developed a 3D cell culture platform termed microbubbles formed in polydimethylsiloxane (PDMS) to mimic the deep cortical unit of the TDLN and co-cultured LN-seeking cancer cells with “super” NK cells to evaluate the therapeutic efficacy of TRAIL-functionalized liposomes (Figure 5E). LNCaP and COLO205 cancer cells, which typically metastasize to lymph nodes in experimental animal models, were co-cultured with “super” NK cells in microbubbles. After 24 h in culture our results showed that “super” NK cells were able to induce apoptosis in cancer cells to a significantly higher degree than endogenous NK cells (Figure 5F) based on propidium iodide (PI) labeling. PI is a fluorescent membrane impermeable DNA binding stain that is a marker for dead cells that have completely lost membrane integrity. Thus, our model showed that lymph node-seeking cancer cells cultured with “super” NK cells in microbubbles could undergo apoptosis after 24 h in culture, validating an adaptive immune therapy based approach to enhance the therapeutic potential of endogenous NK cells. NK cell-mediated immune response is the most straightforward anti-tumor response since it does not involve recognition of a specific TAA on the cancer cell surface. This has led to the development of several NK cell-based immunotherapeutic approaches to target cancer cells in the TDLN [189]. Since TRAIL is a molecule involved in the body’s natural defense mechanisms, it does not produce side effects such as systemic toxicity, which is an issue in therapies that are targeted towards other types of cells in the TDLN such as T-cells and DC functionalized with immunomodulatory cytokines. The cytokines functionalized on liposomes can activate the proliferation of immune cells such as T-cells, which can lead to systemic toxicity due to potentially toxic levels of inflammatory cytokines. Unlike therapies that are targeted towards DC and T-cells that can result in systemic anti-tumor response, TRAIL-liposomes directed to NK cells will primarily target cancer cells in the TDLN. However, it was recently shown that functionalizing liposomes with TRAIL and E-selectin can target cancer cells in the peripheral circulation, eliminating viable cancer cells from the circulation [190].

### 3.5. Therapeutic TRAIL-Liposomes Directed to Circulating Leukocytes

**Figure 6:**
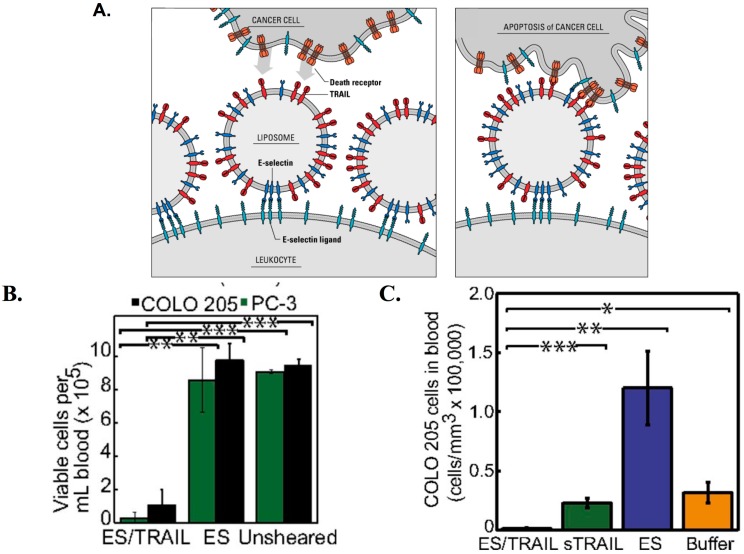
Targeting circulating tumor cells (CTCs) with TRAIL and E-selectin (ES) functionalized liposomes directed to leukocytes: (**A**) Schematic of the “unnatural killer cell” approach. ES/TRAIL liposomes bind to leukocytes expressing E-selectin ligands. Liposome-bound leukocytes (unnatural killer cells) induce apoptosis in cancer cells expressing death receptors during cellular collisions under flow. Adapted from Ref. [189]; (**B**) Number of viable PC-3 and COLO205 cancer cells per mL of blood after treatment with ES/TRAIL or ES liposomes under physiologically relevant shear conditions (188 s^−1^ for 2 h); (**C**) Average number of viable COLO205 cells per mL of blood in mice treated with ES/TRAIL liposomes, soluble TRAIL, ES liposome or buffer injected with fluorescent COLO205 cells. * *p* < 0.01 ** *p* < 0.001, *** *p* < 0.0001 (unpaired *t* test); (**B**,**C**) Reproduced with permission from Ref. [190].

During inflammation, leukocytes in the peripheral circulation reach the affected site by interacting with E- and P-selectin expressed by inflamed endothelium. These adhesive interactions are mediated by E- and P-selectin on activated endothelial cells and selectin ligands on leukocytes [191]. Leukocytes express several ligands for E-selectin such as PSGL-1, ESL-1, CD24 and sialylated carbohydrate ligands [191,192]. A recent review discusses in detail how the extravasation of circulating tumor cells (CTCs) to a secondary site involves adhesive interactions similar to leukocyte extravasation to an inflamed tissue [38,193]. The whole blood microenvironment in which CTCs exist includes several types of leukocytes. The immunosuppressive microenvironment of the TDLN may affect the functions of circulating leukocytes and may explain why certain CTCs escape leukocyte-mediated immune response in blood. Recently, we explored the potential of using nanoscale TRAIL and E-selectin conjugated liposomes to decorate circulating leukocytes for targeting CTCs (Figure 6A) [190]. E-selectin/TRAIL liposomes adhere to multiple leukocyte sub-populations in whole blood under shear flow, forming “unnatural killer cells” with the ability to target CTCs. The compressive force experienced by cells under shear plays an important role in facilitating the receptor-ligand communication. It was found that TRAIL coated leukocytes were able to successfully kill human COLO205 and PC-3 cancer cells in whole blood under physiologically relevant shear rates (Figure 6B). E-selectin/TRAIL liposome treatment also reduced the number of viable cancer cells in the circulation of mice (Figure 6C). Retro-orbital injection of E-selectin/TRAIL liposomes, soluble TRAIL, E-selectin liposomes or buffer followed by tail vein injection of fluorescent COLO205 cells after 30 min significantly reduced the number of viable COLO205 cells in mice treated with E-selectin/TRAIL liposomes (Figure 6C). The rising burden of cancer metastasis has driven a paradigm shift in cancer research toward more targeted therapeutic approaches. Conventional broad-spectrum radiotherapy and chemotherapy may have deleterious effects in patients, drastically reducing patient quality of life. Hence, there is growing interest in the development of more targeted therapeutic approaches to interrupt critical steps in the metastatic cascade. Developing therapies to target cancer cells in the peripheral circulation can help to reduce the metastatic spread of cancer. Given the ability of circulating leukocytes to home to the TDLN through the HEV, receptor-targeted/TRAIL liposomes could potentially be used to attack cancer cells in the TDLN.

## 4. Concluding Remarks

In 1909, Paul Ehrlich predicted that activation of the host immune system could target cancer cells. This prediction led to a century of speculation about utilizing the immune system to fight cancer since it cannot attack “self” cells. Tumor cells are primarily host cells with unlimited growth, but owing to the mutations and altered gene expression, they are nevertheless different from normal cells. The discovery of TAA by Pierre van der Bruggen and his colleagues in 1991 represents a milestone in tumor immunotherapy leading to a class of therapies targeted towards TAA. Recent advancements in engineering and biocompatible drug delivery systems have catalyzed the interest in tumor immunotherapy. The ability of nanoscale liposomes to target the TDLN and activate systemic anti-tumor immune response could potentially be used to diminish the metastatic burden in cancer patients. Liposome-based cancer vaccines targeting the TDLN have promising potential to deliver therapeutic agents to kill cancer cells in the TDLN. The immunosuppressive microenvironment of the TDLN allows cancer cells to form micrometastases that can remain dormant for several years before forming overt metastatic lesions. Although most existing TDLN targeted therapies activate the host immune system to elicit an anti-tumor response, there is growing interest in the development of therapies that deliver drugs to the TDLN in order to kill cancer cells. The ability to engineer nanoscale liposomes offers myriad possibilities. Liposomes could be engineered to deliver molecules that can trigger the host immune response and also deliver therapeutic agents to kill cancer cells in the TDLN to reverse the immunosuppressive microenvironment that favors metastatic progression. Clinically, we envision that TDLN-directed nanoscale liposomes containing therapeutic drugs and immunomodulatory cytokines could be used to interfere with the lymphatic spread of cancer. It is important to design appropriate therapeutic dosage and develop screening methodologies to study the biodistribution of nanoscale liposomes. Although we are still far from the clinical use of TDLN-directed liposomes, liposome-based approaches will play an important role in therapeutic oncology in the near future.
